# Effect of Anatase Synthesis on the Performance of Dye-Sensitized Solar Cells

**DOI:** 10.1186/s11671-015-0991-3

**Published:** 2015-07-29

**Authors:** Mario Alberto Sánchez-García, Xim Bokhimi, Arturo Maldonado-Álvarez, Antonio Esteban Jiménez-González

**Affiliations:** Instituto de Física, Universidad Nacional Autónoma de México (IF-UNAM), Apartado Postal 20-364, México, D.F. 04510 México; Instituto de energías Renovables (IER-UNAM), Temixco, Morelos 62580 México; CINVESTAV-IPN Unidad Zacatenco, Sección de Electrónica del Estado Sólido, México, D.F. 07360 México

**Keywords:** High-pressure semiconductors, Hydrothermal synthesis, Mesoporous anatase films, Dye-sensitized solar cells

## Abstract

Anatase nanoparticles were synthesized from a titanium isopropoxide solution using a hydrothermal process at different pressures in an autoclave system while keeping the volume of the solution constant. As the autoclave pressure was increased from 1 to 71 atm (23 to 210 °C), the crystal size in the nanoparticles increased from 9 to 13.8 nm. The anatase nanoparticles were used to build dye-sensitized solar cells (DSSC). Mesoporous films of this oxide were deposited over conducting SnO_2_:F substrates using the screen-printing technique and then annealed at 530 °C at 1 atm of air pressure. The morphology of the mesoporous film surface of anatase, studied using scanning electron microscopy, revealed that the crystal size and pore distribution were functions of the pressure conditions. The energy band gap of the films as a function of the crystal size exhibited quantum effects below 11.8 nm. The effects of the anatase synthesis conditions and properties of the mesoporous film on the DSSC-type solar cell parameters, η%, *V*_OC_, *J*_SC_, and FF, were also investigated: the mesoporous anatase films prepared at 200 °C (54 atm of pressure in the autoclave) and annealed at 530 °C in air generated the best solar cell, having the highest conversion efficiency.

## Background

Titanium dioxide (TiO_2_) is a width band gap semiconductor oxide with a wide spectrum of physical and chemical properties, among which we highlight the electrical conductivity, photosensitivity, photovoltaic activity, and chemical stability in acidic and aqueous environments. These features make this material an appropriate candidate for a large variety of potential applications. TiO_2_ is observed in the polymorphic phases rutile, brookite and anatase, with the last one being the most widely used in applications such as photocatalysis, anticorrosive coatings, antireflection films, and solar cells [1–3]. This crystalline phase exhibits photosensitivity factors *S*(= *σ*_light_ − *σ*_dark_/*σ*_dark_) on the order of 10^4^, where *σ*_light_ and *σ*_dark_ represent the electrical conductivity under light and in the dark, respectively. The sensitivity factor S ~ 10^4^ tells us that the system is quite capable of absorbing electromagnetic radiation in the ultraviolet–visible (UV–Vis) range below 370 nm and of generating electron–hole pairs. In a process of charge carrier photogeneration, these capabilities are necessary for adequate performance in applications such as solar cells, diodes, and photocatalysis.

Because of its structural configuration consisting of layers and because a liquid-phase electrolyte is used, dye-sensitized solar cells constitute an electrochemical cell. These cells use an n-type TiO_2_ semiconductor in the anatase phase in the blocking layer (compact layer) as well as in mesoporous layer forms. The blocking layer (TiO_2_^b^), with a thickness of 100 nm, can be prepared through sol–gel chemical deposition techniques, spin coating, and atomic layer deposition (ALD). The objective of this film is to block direct contact between the redox pair and the surface of the conducting glass, avoiding the construction of trap sites in the interface and reducing electronic recombination, which increases the efficiency of solar cells. Passivation through the compact layer also has the advantage of reducing series resistance of the solar cell, as it improves contact between the mesoporous layer and the conducting glass.

The mesoporous (TiO_2_^m^) film plays a fundamental role in the design of solar cells when they are sensitized with dyes [4], quantum dots [5], or perovskites [6] because it plays the role of the n-type semiconductor in the p–n heterojunction while the sensitizers play the role of the “p-type semiconductor.” The mesoporous anatase film in the structure of sensitized solar cells has the purpose of absorbing the sensitizer (dye, quantum dots, or perovskites); therefore, it is convenient for this film to have a large surface area to absorb as much sensitizer as possible, which can be achieved using a mechanically stable mesoporous film with a pore size below 50 nm [7, 8].

Several techniques exist to prepare mesoporous anatase films from TiO_2_ nanoparticles synthesized in the laboratory such as spin-coating, doctor-blading, spray pyrolysis, and screen-printing techniques [8–10]. In addition, several procedures have been developed to synthesize TiO_2_; among them, the hydrothermal process using an autoclave has been an effective method to obtain crystal sizes between 6 and 15 nm at pressure and temperature values above 50 atm and 200 °C, respectively [11, 12].

**Fig. 1:**
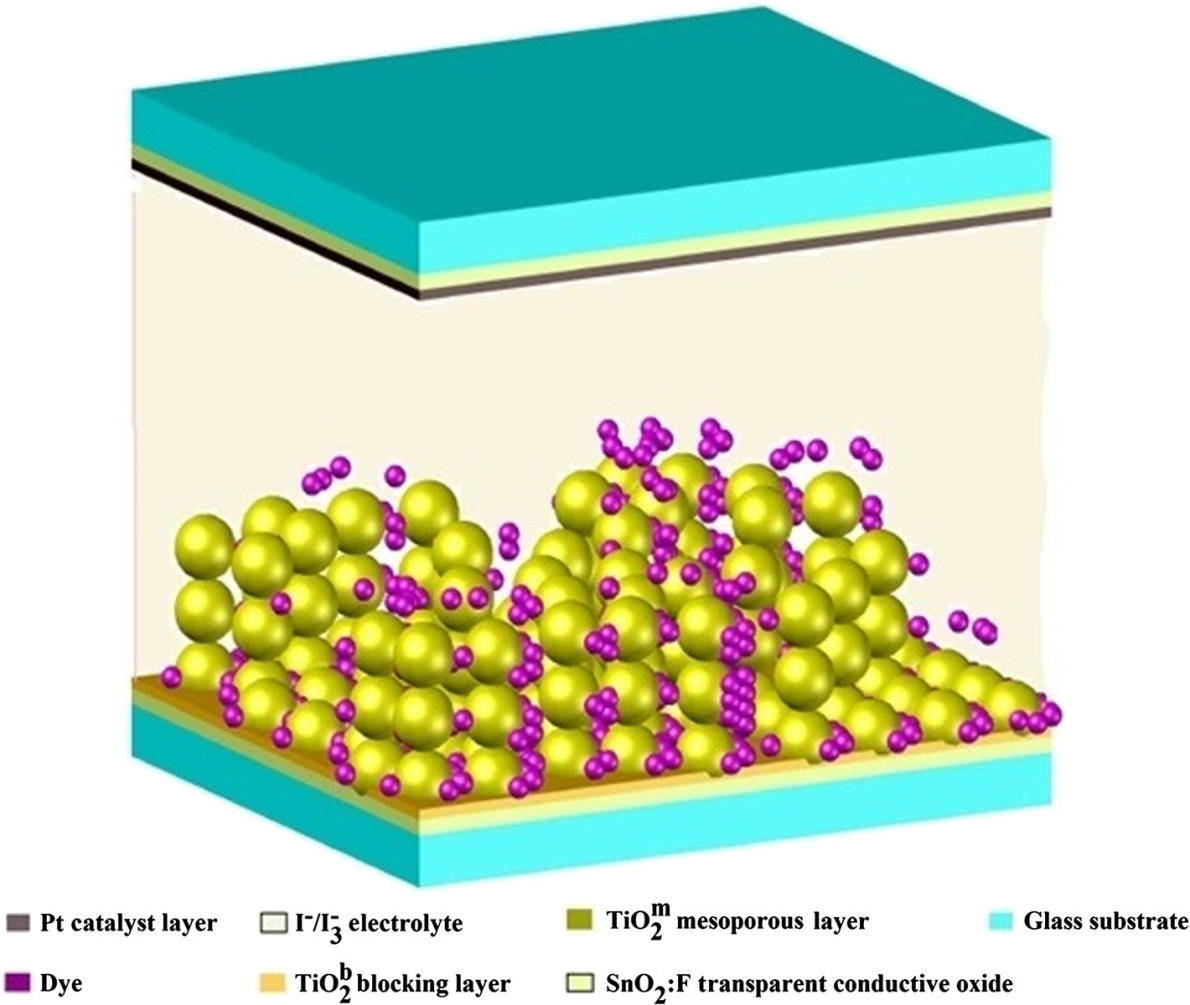
Structure of a dye-sensitized solar cell

Figure 1 shows the structure of a sensitized solar cell. The lower part of the figure shows the glass substrate, with its top surface coated with a transparent conductive oxide (TCO) of SnO_2_:F. A blocking TiO_2_^b^ layer is deposited over the TCO, and the mesoporous TiO_2_^m^ layer is grown on top of that layer.

The mesoporous TiO_2_^m^ system is subsequently sensitized with dye N-719. The SnO_2_:F/TiO_2_^b^/TiO_2_^m^ arrangement sensitized with the dye constitutes the working electrode, also known as the active electrode in the dye-sensitized solar cell (DSSC). The top part of Fig. 1, facing downward, shows a glass substrate coated with a thin platinum film, which constitutes the counter electrode, with the purpose of regenerating the electrolyte. The intermediate space between the active electrode and counter electrode is filled with the electrolyte, which contains the I_3_^−^/I^−^ redox pair.

When the DSSC cell is illuminated, the dye molecules absorb the electromagnetic radiation, and for each photon absorbed, an electronic transition is generated from its basic state (S^o^) toward its excited state (S*), that is, the electron goes from the HOMO to the LUMO level in the dye, and from this state, the electron is injected into the conduction band of the semiconductor oxide, generating a dye cation (S^+^). Therefore, it is necessary to regenerate the dye, for which an electron is taken from the iodides (I^−^) contained in the electrolytic solution, resulting in their oxidation and formation of triiodide (I_3_^−^), which in turn is reduced by taking electrons from the counter electrode to then return to the original state (I^−^) [13].

Because of the importance of the mesoporous TiO_2_^m^ layer in the construction and operation of DSSC cells and because its properties depend on the size of the crystal, this study emphasizes the synthesis of mesoporous TiO_2_ nanoparticles. The synthesis was performed using a hydrothermal treatment, in which the solution temperature, and therefore its pressure within an autoclave, was increased while keeping the volume constant (300 ml). The titanium oxide nanoparticles obtained using this method were used to prepare a paste for serigraphic printing of mesoporous anatase films of the n-type semiconductor. The structural and optical properties as well as the energy levels and electrical properties of the mesoporous TiO_2_^m^ layer were investigated using X-ray diffraction (XRD), field-emission scanning electron microscopy (FE-SEM), UV–Vis spectrophotometry, and electrical characterization.

Based on the experimental results obtained for the mesoporous TiO_2_^m^ system, we studied the correlation between the quantum effect observed in the nanoparticles with crystal sizes below 11.8 nm and the experimental variables of the synthesis of the mesoporous system, namely pressure and temperature.

Using the pastes for serigraphic printing as described previously, DSSC-type solar cells were fabricated to study the effect of the pressure and temperature conditions of the hydrothermal mesoporous semiconductor synthesis on the experimental parameters that characterize the solar cells such as the conversion efficiency (η%), open circuit voltage (*V*_OC_), short-circuit current (*J*_SC_), and fill factor (FF).

## Methods

### Preparation of the TiO_2_^b^ Blocking Film Using the Sol–Gel Technique

To fabricate the TiO_2_^b^ blocking layer, a precursor solution of titanium isopropoxide, deionized water, hydrochloric acid, and ethanol was prepared in an air environment at atmospheric pressure. In a Florence flask, 2.5 ml of titanium isopropoxide and 119 ml of ethanol were mixed. Then, a solution was added dropwise to the initial mixture that contained deionized water (5 ml), hydrochloric acid (3.75 ml), and ethanol (119.35 ml). The solution remained agitated for 24 h [13, 14]. The blocking layer was obtained by submerging and extracting transparent TEC15 conducting substrates (SnO_2_:F) in the precursor solution at a speed of 1.5 × 10^−3^ m/s. Then, the film of the titanium complex adhered to the SnO_2_:F substrate was subjected to a thermal treatment at 130 °C in air for 3 min to transform it into titanium hydroxide. These steps correspond to an immersion-heating cycle. To reach a thickness of approximately 100 nm, four immersion-heating cycles were required, with a final treatment at 400 °C for 1 h in air at atmospheric pressure.

### Synthesis of TiO_2_ Nanoparticles

The experimental procedure followed for the synthesis of TiO_2_ nanoparticles at high pressure and temperature was similar to that reported in [9]. A closed cylindrical Teflon-lined stainless autoclave was used in the following procedure: a mixture was made of 21.2 ml of titanium isopropoxide and 4 ml of acetic acid, which was agitated at 300 rpm for 15 min at room temperature. This mixture was added dropwise to 145 ml of deionized water, maintaining agitation at 500 rpm. Then, the agitation velocity was increased to 700 rpm for 1 h. Then, 1.4 ml of nitric acid was added to this mixture. After adding the nitric acid, the temperature was increased to 80 °C, and these conditions were maintained for 45 min. Then, the solution was peptized at 5 °C for 75 min. Finally, 150 ml of the mixture was introduced into the autoclave to perform the hydrothermal process at temperatures between 127 and 210 °C for 12 h. After completion of the reaction, the autoclave was cooled down to room temperature. The resulting nanoparticles were washed and centrifuged with ethanol and acetone to eliminate any acidic residues.

### Preparation of the Paste for Serigraphic Printing of the Mesoporous TiO_2_^m^

Preparation of the mesoporous TiO_2_^m^ paste was performed in the following manner: once the TiO_2_ nanoparticles were centrifuged, the acetone-moistened nanoparticles were weighed. For each gram of acetone-moistened TiO_2_ nanoparticles, a mixture of 2 g terpineol was prepared in a flask, while in another flask, 0.075 g ethyl cellulose was mixed with anhydrous ethanol. The terpineol was used as a dispersing agent for the TiO_2_ nanoparticles in the paste, whereas the ethyl cellulose was added to the mixture to provide a porous structure to the film. Both mixtures separately received an ultrasonic treatment.

The ethyl cellulose mixture with ethanol was agitated for enough time for the mixture to completely fuse in the ethanol. Analogously, the TiO_2_ nanoparticle mixture with terpineol was agitated enough for no TiO_2_ aggregations to be present in the terpineol. After the ultrasonic treatment, the ethyl cellulose solution was added to the TiO_2_ and terpineol mixture, which received an ultrasonic treatment for 15 to 30 min. Subsequently, the latter mixture was introduced into a rotavapor to evaporate the excess ethanol and acetone. The procedure in the rotavapor was performed at 40 °C and at a vacuum pressure of −0.7 bar for approximately 3 h. Finally, the paste obtained was used to prepare the mesoporous TiO_2_^m^ films.

### Preparation of the SnO_2_:F/TiO_2_^b^/TiO_2_^m^ Heterojunction

The procedure to fabricate the SnO_2_:F/TiO_2_^b^/TiO_2_^m^ heterojunction as the active electrode of the DSSC was as follows: using the sol–gel technique, the compact TiO_2_^b^ film was first deposited on the SnO_2_:F, and on top of that, the mesoporous TiO_2_^m^ serigraphic printing was performed using the paste described in the previous section. The serigraphic printing of the mesoporous TiO_2_^m^ films involved the use of manual screen printing with a 140-T mesh (140 strands/cm) and a deposit area capacity of 1 × 0.5 cm^2^. The thickness of the film was controlled based on the number of layers to be deposited through this technique, which provides an approximate thickness of 12 μm when 18 prints are performed.

After depositing each layer using the screen-printing technique, the porous films received a thermal treatment at 130 °C for 3 min. Once all the screen printing was performed, the temperature was increased in 100 °C intervals with rest periods of 10 min between each increase. After reaching 530 °C, the mesoporous anatase film was maintained at this temperature for 1 h.

To prevent the film from “exploding” because of “violent” evaporation of the organic compounds (ethyl cellulose and terpineol), in the ninth layer, the films received an average thermal treatment at 400 °C for 1 h. After the thermal treatment at 530 °C, the mesoporous TiO_2_^m^ films were allowed to cool down until they reached 80 °C and were then submerged in a 0.5 mM solution of N-719 dye in ethanol for 20 h in order sensitize the films. The electrodes obtained were washed in ethanol and dried with nitrogen before assembling the DSSC cell.

### Preparation of the DSSC Counter Electrode

Starting with a 40 mM solution of chloroplatinic acid in isopropyl alcohol, the platinum catalyzer was deposited with a brush over a transparent SnO_2_:F conductor, to which a thermal treatment was later applied at 130 °C in air for 3 min with the purpose of evaporating the solvent. A total of three layers of platinum were deposited. Finally, this counter electrode was subjected to a thermal treatment at 400 °C in air for 10 min [9].

### The Electrolytic Solution

The electrolytic solution that contains the redox pair I_3_^−^/I^−^ was prepared using 1-methyl-3-propyl-imidazolium iodide (PMII) (0.6 M), guanidine thiocyanate (GuSCN) (0.1 M), lithium iodide (LiI) (0.1 M), 4-tert-Butylpyridine (TBP) (0.5 M), and iodine I_2_ (0.05 M) in acetonitrile (85 %) and valeronitrile (15 %); these concentrations are based on the solutions reported in [15–18]. The electrolyte was introduced in the DSSC cell through two holes in the counter electrode, which were subsequently sealed.

### Experimental Set-up

A Shimadzu 2100 UV–Vis spectrophotometer was used to measure the optical transmittance and reflectance of the TiO_2_ thin films. The X-ray diffraction (XRD) analysis was performed using Cu K_α_ radiation (*λ* = 1.54056 Å) at 40 kV and 30 mA with a Rigaku RINT 2200VK/PC diffractometer. The crystallite size of the films was calculated using the Scherrer formula (*d* = 0*.*94*λ/B*(2*θ*) cos *θ*), where *λ* is the wavelength of the X-rays and *B*(2*θ*) is the full width at the half-maximum intensity (FWHM) of the analyzed peak with the maximum intensity at the angle 2*θ* [19]. The surface morphology of the blocking TiO_2_^b^ and mesoporous TiO_2_^m^ samples was analyzed using scanning electron microscopy with a Hitachi FE-SEM S-5500 microscope. *I* vs. *t* and *I* vs. *V* curves of the solar cells were obtained using a Keithley 238 voltage/current measurement and supply device controlled using LabVIEW, which was also used to obtain the data. A halogen lamp ELH300W at 127 V calibrated to 1 sun (100 mW cm^−2^) by means of a MP3-25 solar panel was used as the excitation source for the DSSC solar cells. Photoresponse (*I* vs. *t*) curves have three periods of measurement: (i) darkness (60 s), (ii) illumination (120 s), and (iii) darkness (120 s).

## Results and Discussion

### TiO_2_^b^ Blocking Film

**Fig. 2:**
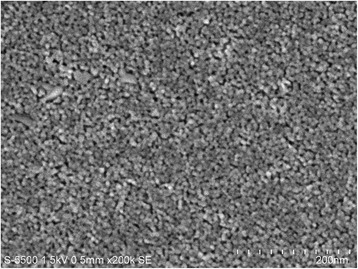
FE-SEM micrograph of the fabricated TiO_2_ blocking film

Figure 2 shows a micrograph acquired from secondary electrons of the blocking layer. Its compact morphology can be observed, which is essential to avoid undesirable recombination processes caused by direct contact between the electrolyte and the SnO_2_:F.

**Fig. 3:**
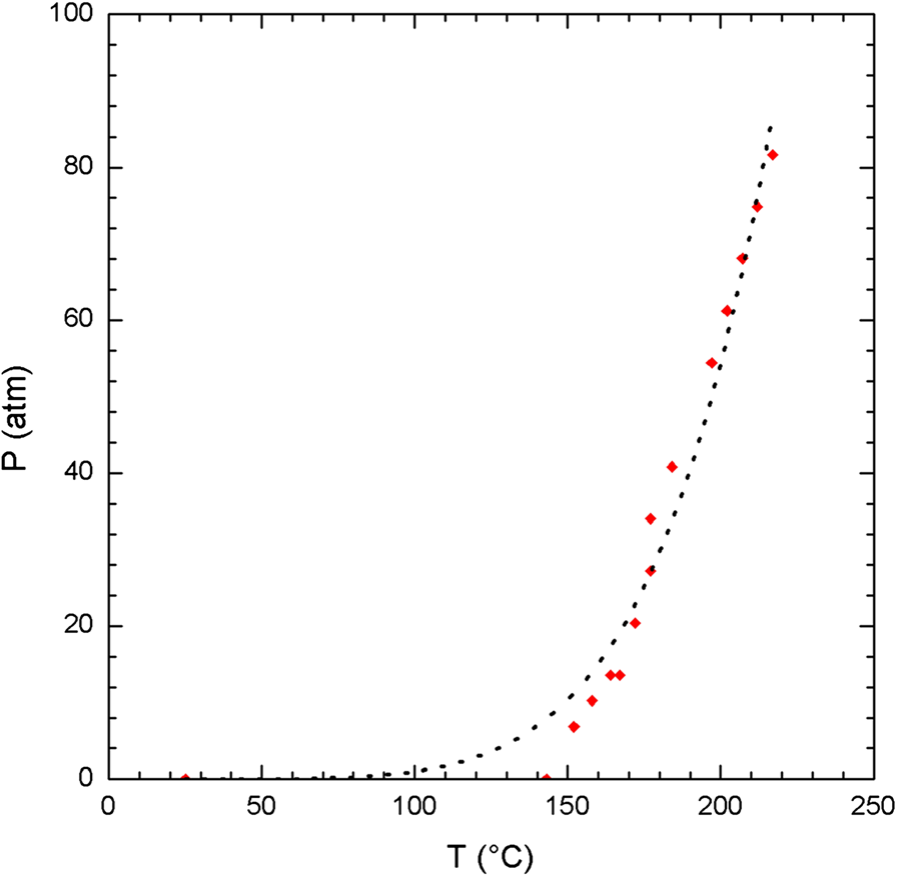
*P* vs. *T* curve (*red dots*) and allometric adjustment (*discontinuous black line*) calculated based on experimental conditions in the autoclave during the hydrothermal process

Figure 3 shows the behavior under pressure inside the autoclave as a function of temperature during the hydrothermal process to synthesize the TiO_2_ nanoparticles, which were used when preparing the paste for screen printing of the mesoporous TiO_2_^m^ layer. It can be observed from the *P* vs. *T* curve in Fig. 3 that for temperatures lower than 150 °C, the pressure falls below 7 atm. At 100 °C, the system reaches a pressure near 1 atm, and for temperatures above 150 °C, the pressure rapidly increases until it reaches a value of 81 atm at 210 °C. The abrupt growth in pressure inside the autoclave plays a fundamental role in the finite growth of the nanoparticle, as will be explained in detail in the following section. An allometric adjustment, represented in Eq. 1, allowed for adjusting of the approximate curve to the experimental data (black dot curve in Fig. 3), which is very similar to the vapor pressure curves of ammonia, water, and mercury [20].

1 $$ P\left(\mathrm{atm}\right)=2.928 \times {10}^{-12}{\left[T\left({}^{\circ}\mathrm{C}\right)\right]}^{5.767} $$

Using this autoclave, the synthesis of TiO_2_ nanoparticles was performed at working temperatures of 127, 148, 167, 182, 200, and 210 °C.

**Fig. 4:**
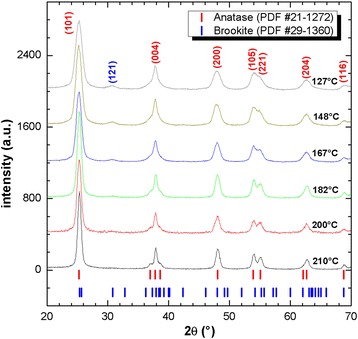
XRD patterns of the synthesized anatase nanoparticles

Figure 4 shows the X-ray diffraction patterns of the TiO_2_ nanoparticles synthesized in the autoclave at temperatures of 148, 167, 182, 200, and 210 °C. For temperatures below 200 °C, the phases encountered were anatase (PDF 21-1272) and brookite (PDF 29-1360), whereas for temperatures of 200 °C or higher, only the anatase phase was encountered. For all the temperatures, the predominant crystalline phase was anatase, for which the FWHM of the highest intensity peak at (101) decreased as the synthesis temperature increased, which indicates a growth in the crystal size. The crystal size of the anatase nanoparticles as it relates to temperature and pressure during the synthesis process is described in Table 1, where the pressure values were obtained based on Eq. 1. It is possible to observe in Table 1 that as the temperature increases, the pressure also increases within the autoclave, while at the same time, the crystal size becomes bigger.

**Table 1:** Crystallite size as a function of temperature and pressure conditions in the autoclave

Synthesis temperature (°C)	Synthesis pressure (atm)	Crystallite size (nm)
127	4	9
148	9.5	9.5
167	19.5	10
182	31.7	12
200	54	12
210	71	13.8

**Fig. 5:**
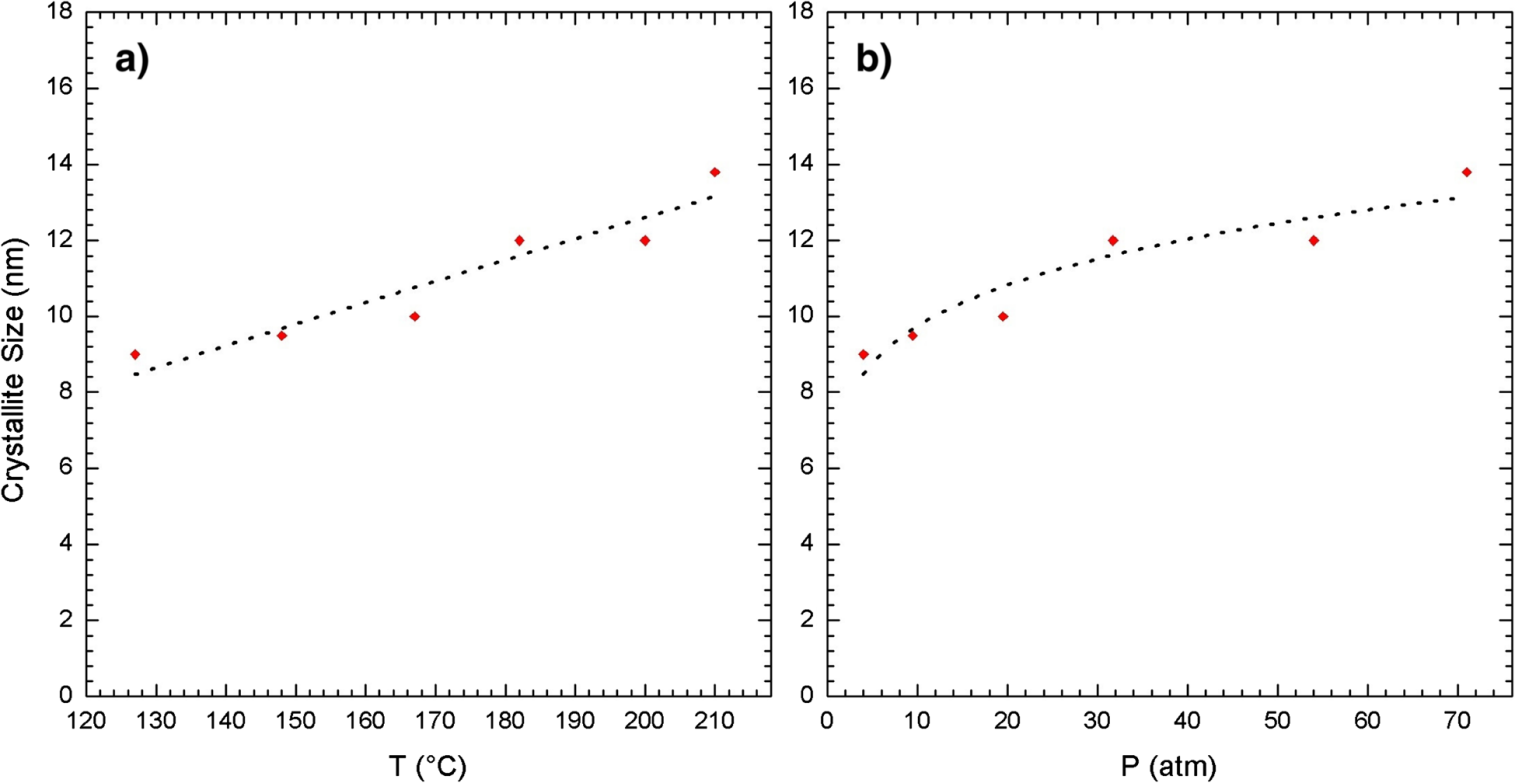
**a** Crystallite size vs. *T* and **b** crystallite size vs. *P* (*red dots*) in addition to linear and allometric adjustment (*black discontinuous line*) of the TiO_2_ nanoparticles synthesized in the autoclave

From Fig. 5a, it is possible to observe that as the temperature increases in the autoclave, the crystal size increases linearly. However, Fig. 5b demonstrates that the relationship between the crystal size and pressure is not linear, which is a very important result, meaning that the pressure in the autoclave is an experimental variable that bounds the crystal size and its geometric configuration and prevents greater aggregation of the nanoparticles, as will be observed in the electron microscopy results. Figure 5b also shows an allometric adjustment (partial black line) of the crystal size *d* represented by Eq. 2.

2 $$ d\left(\mathrm{nm}\right)=6.865{\left[P\left(\mathrm{atm}\right)\right]}^{0.152} $$

These results indicated very small growth in the nanoparticles as a function of pressure inside the autoclave, where the smallest crystal size obtained was 9 nm at 4 atm (127 °C), and the largest one obtained was 13.8 nm at 71 atm (210 °C).

### Analysis of the Mesoporous TiO_2_^m^ Films

Using the nanoparticles synthesized in the “TiO_2_^b^ Blocking Film” section, pastes were fabricated for the screen printing of mesoporous TiO_2_. These pastes were labeled according to the synthesis temperature of the TiO_2_ as A127, A148, A167, A182, A200, and A210. The number to the right of this notation indicates the synthesis temperature

**Fig. 6:**
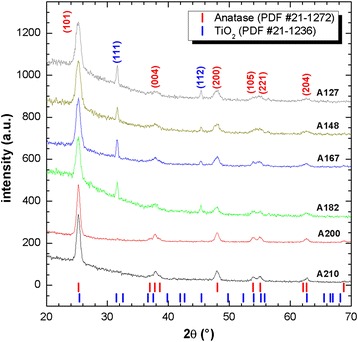
XRD patterns of the mesoporous TiO_2_^m^ films annealed at 530 °C

As explained in the “Preparation of the SnO_2_:F/TiO_2_^b^/TiO_2_^m^ Heterojunction” section, after screen printing, the mesoporous TiO_2_^m^ films were thermally treated at 530 °C. Figure 6 shows the X-ray diffraction patterns of the mesoporous TiO_2_ films fabricated using these pastes. One can observe that the anatase crystalline phase was maintained in all cases and that an additional TiO_2_ phase identified by PDF 21-1236 with (111) and (112) reflections was present in the samples fabricated with pastes A127, A148, A167, and A182, although that phase disappears at temperatures above 182 °C.

**Fig. 7:**
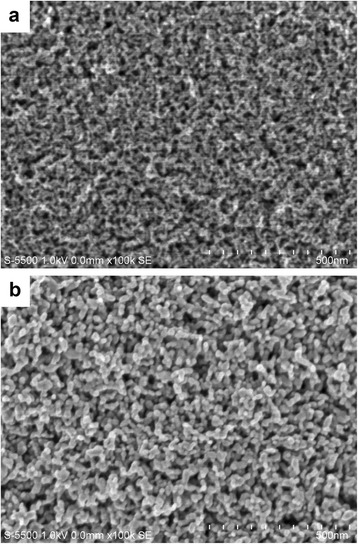
Micrographs of TiO_2_ porous films fabricated with paste: **a** A127 and **b** A210

Figure 7 shows the surface morphology observed using scanning electron microscopy of the mesoporous anatase films fabricated using screen printing and thermally treated in air at 530 °C. For sample A127 showed in Fig. 7a, one can observe a uniform TiO_2_ nanoparticle film with little aggregation and with an average pore size of 23.93 nm, whereas sample A210, as shown in Fig. 7b, exhibits a slightly increased crystal size, some aggregation of the TiO_2_ particles, and the average pore size remains the same. Using the software QUARTZ PC integrated with the FE SEM S-5500 equipment, it was possible to determine the particle size and pore size distribution of each film, as described in Table 2. It can be observed that the particle sizes determined by scanning electron microscopy in the TiO_2_^m^ mesoporous films were slightly greater than the crystallite sizes obtained by X-ray diffraction, although the trend of the crystal size growth observed for the FE-SEM images and XRD was the same. This difference can be caused by the presence of microstrain in the crystallites which was not taken into account during the calculations of crystallite sizes. Crystallite size values will be larger when this microstrain is considered.

**Table 2:** Crystallite and pore sizes and direct and indirect band gap values for mesoporous TiO_2_ films prepared with pastes A127 to A210 and subsequently treated thermally at 530 °C in air

Sample	Synthesis temperature in the autoclave (°C)	XRD crystallite size *d* (nm)	FE-SEM pore size (nm)	Direct band gap Eg^dir^ (eV)	Indirect band gap Eg^ind^ (eV)
A127	127	10.8	23.964 ± 2.55	3.69	3.52
A148	148	11	26.36 ± 6.41	3.48	3.40
A167	167	11.8	25.04 ± 3.41	3.45	3.34
A182	182	12.3	21.354 ± 3.00	3.44	3.34
A200	200	12.4	23.116 ± 4.23	3.44	3.34
A210	210	14.1	21.407 ± 2.29	3.44	3.34

Table 2 shows that the pore size of each of the mesoporous TiO_2_ films ranges from 20 to 30 nm, with the values always being smaller than 50 nm. Therefore, these TiO_2_ films can be considered mesoporous according to the IUPAC rules [21].

TiO_2_ in its anatase crystalline phase has an indirect prohibited energy band Eg^ind^ = 3.2 eV [2]. To calculate the energy band gap of a semiconductor, there is a general expression that relates the absorption coefficient to the energy band gap, which is given as (*αhν*)^*m*^ versus (*E* − *hν*), where *m* is an integer or semi-integer, *h* is the Planck constant, *hν* is the energy of the electromagnetic radiation, and *E* is the energy of the involved quantum levels in the semiconductor [22, 23]. When (*αhν*)^*m*^ = 0 for certain *ν*_0_, then *E* = Eg = *hν*_0_.

When *m* = 2, the semiconductor has a direct band gap, and under electromagnetic excitation, the electron executes a direct allowed transition from the valence to the conduction band, whereas when *m* = 1*/*2, the semiconductor has an indirect band gap, and the transitions are indirectly allowed. The absorption coefficient α for a semiconductor material is described through the mathematical expression $\alpha =\frac{1}{t} ln\left(\frac{100-R\%}{T\%}\right)$, which is obtained based on the optical transmittance *T* and reflectance *R* of the TiO_2_ semiconductor, where *t* is the film thickness.

Table 2 lists the crystal size of the mesoporous TiO_2_^m^ films labeled A127 to A210 and thermally treated at 530 °C as well as the corresponding values for the direct and indirect band gap. The values for the direct and indirect band gaps decrease as the particle size of the mesoporous TiO_2_^m^ films increases; the crystallite size of 10.8 nm corresponds to the highest value of the band gap, both direct and indirect, that is, 3.69 and 3.52 eV, respectively.

**Fig. 8:**
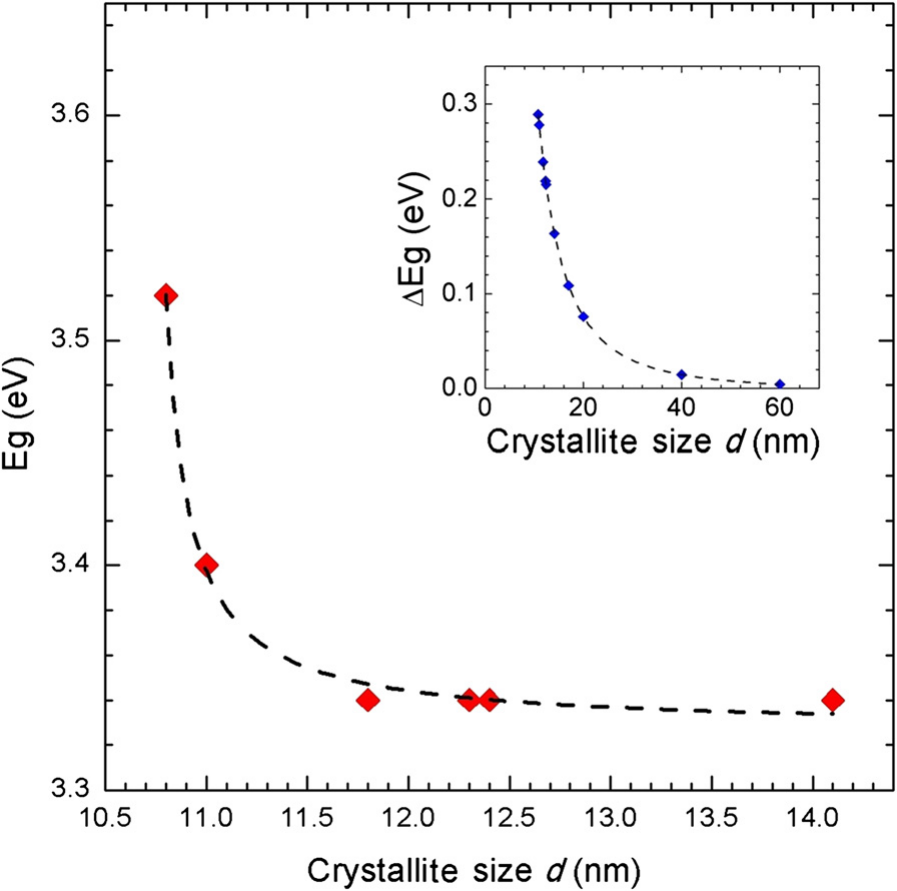
Value of the indirect energy band gap (*red diamonds*) of the mesoporous TiO_2_^m^ films as a function of the crystallite size. The insert shows an approximation of the indirect band gap Eg^ind^ of the mesoporous TiO_2_^m^ according to Eq. 3

Figure 8 shows a curve of the energy band gap for the indirect transitions allowed for mesoporous TiO_2_^m^ films as a function of the crystal size, which was prepared from the data presented in Table 2. For crystallite sizes below 11.8 nm, the energy band gap abruptly grows, which corresponds to a quantum effect by reducing the crystal size of the mesoporous TiO_2_^m^ films. For crystallite sizes greater than 11.8 nm, the indirect prohibited band value Eg^ind^ tends toward 3.34 eV. The small difference in the prohibited band value observed in our films compared with those reported in the literature (Eg = 3.2 eV) is explained by the fact that the crystallite size remains small (14.1 nm); to approach the values reported in the literature, the size must increase.

In general, the quantum effect observed in the band gap relationship with the crystallite size in semiconductor nanomaterials can be represented by the equation

3 $$ \varDelta \mathrm{Eg}\left(\mathrm{eV}\right)=\mathrm{Eg}\left(\mathrm{eV}\right) - {\mathrm{Eg}}_{\mathrm{b}} = \frac{h^2}{2\ {d}^2}\left[\frac{1}{m_{\mathrm{e}}}+\frac{1}{m_{\mathrm{h}}}\right]-\frac{1.8{e}^2}{\in\ r\ } $$

which provides the energy shift *Δ*Eg(eV) experienced by the energy band when the crystal size *d* becomes too small (*d* < 11.8 nm) relative to the bulk band gap Eg_b_, where *h* represents the Planck constant, *r* is the radius of the crystallite (*d =* 2*r*), *m*_e_ is the effective mass of the electron, *m*_h_ is the effective mass of the hole, *ε* is the dielectric constant of the material, and *e* is the electric charge of the electron (1.6022 × 10^−19^ C) [24].

**Fig. 9:**
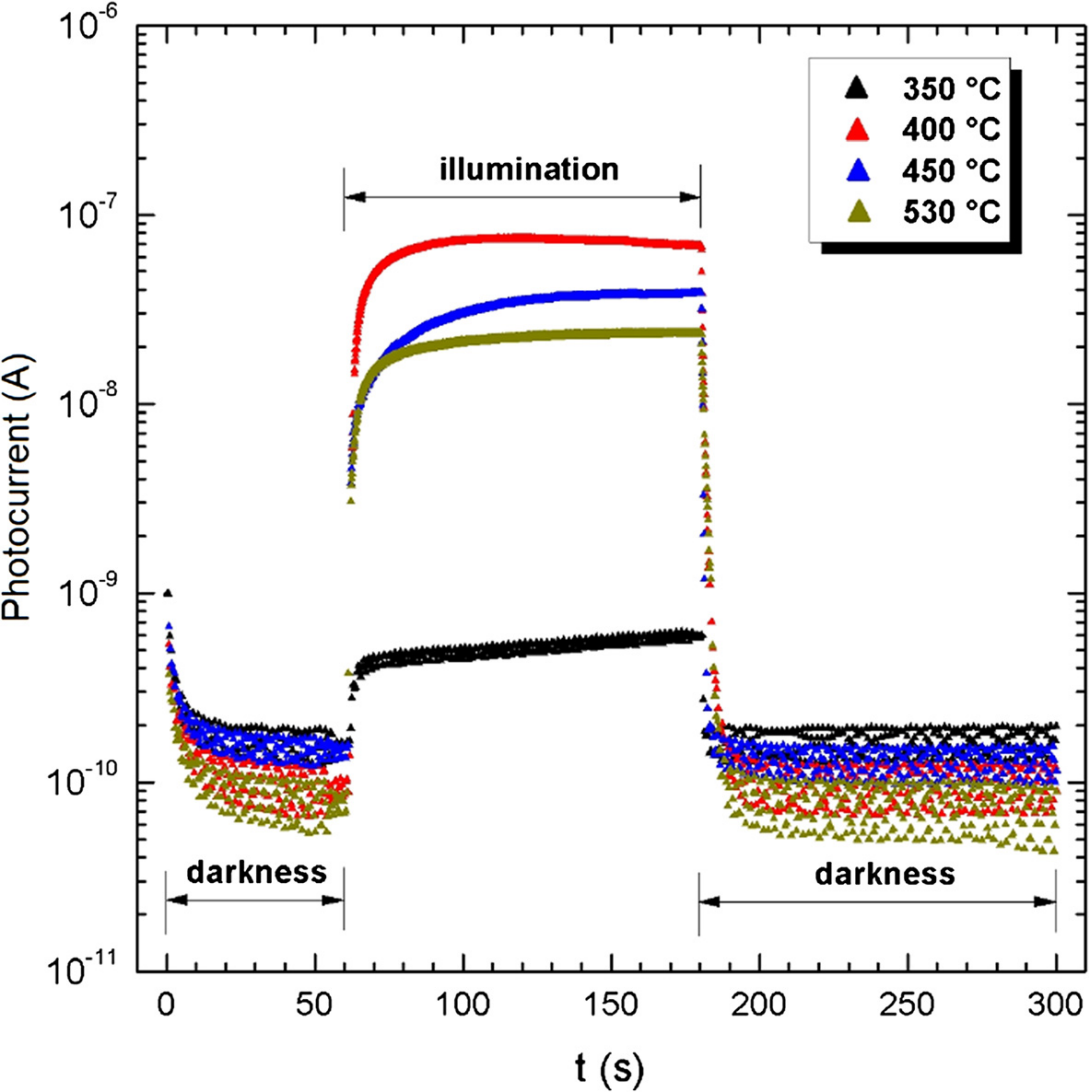
Photoresponse curves of mesoporous TiO_2_^m^ fabricated with paste A200

Considering an anatase crystalline phase for the TiO_2_^m^ and the numerical values of the magnitudes expressed in Eq. 3 reported in the literature [24, 25], that is, *h* = 4.14 × 10^−15^eVs, *e*^2^ = (1.6022 × 10^−19^ C)^2^, *m*_e_ = 9.11 × 10^−31^ Kg, *m*_e_* = 0.8*m*_e_, *m*_h_* = 0.2*m*_e_, *e* = 180, and Eg_b_ = 3.2 eV, and the crystallite sizes *d* indicated in Table 2, the insert in Fig. 9 outlines the energy shift *ΔE*(eV) that the indirect energy band gap experiences, as expressed through Eq. 3, with a reduction in crystal size. In total, an average widening of the energy band gap of 0.12 eV is recorded relative to the bulk value when the crystallite size is reduced from 12.4 to 10.8 nm. The *ΔE*(eV) shifts reported here are very similar in the order of magnitude to those reported in the literature.

Enright synthesized TiO_2_ nanocrystals (from 2 to 9 nm) with an anatase crystalline phase using the electrodeposition method using TiCl_4_ as a precursor reactant [24]. Using spectroelectrochemical and spectrophotometric techniques applied to transparent nanocrystalline semiconductor electrodes, it was possible to determine the energy values of the valence and conduction band edges, effective mass of the electron, effective mass of the hole, and energy shift *Δ*Eg(eV) = 0.33 eV for the indirect prohibited band when the crystal size decreased [25]. For photocatalysis applications, Lee and collaborators, based on a titanium isopropoxide solution as the precursor reactant, synthesized mesoporous TiO_2_ with the anatase crystalline phase (Eg = 3.2 eV) supported on zeolites SBA-15 and MCM-41. In these systems, these researchers were able to determine a shift in the energy band gap *Δ*Eg(eV) = 0.1 eV when the crystal size varied from 2.6 to 12 nm [26]. Hegasy and collaborators synthesized TiO_2_ with the anatase phase based on a gel prepared in a titanium n-propoxide solution (Ti(O-n-Pr)_4_, formamide, HCL, and H_2_O). These researchers observed crystal sizes between 2.6 and 12 nm in samples subjected to thermal treatment at 400 °C. Following the results of Enright, Hegasy was able to follow the evolution of the indirect energy band gap as a function of the anatase crystal size and observed an energy shift of that band *Δ*Eg(eV) = 0.15 eV. By applying TiO_2_^m^ with the anatase phase to the development of DSSC solar cells, Hegasy and collaborators reported an efficiency *η* = 0.68 % [27].

Figure 9 shows photoresponse curves of a mesoporous TiO_2_^m^ layer deposited (4.7-μm thick) on corning glass by screen printing with the paste labeled as A200 and treated in air at different temperatures. Starting with a temperature treatment of 350 °C, below this temperature TiO_2_ is not photoconductive, the photocurrent signal increases not only with the temperature but also with the crystallite size. When the temperature reaches 400 °C, a photocurrent value of 7 × 10^−8^ A is achieved. Over 400 °C, the photocurrent starts to decrease, and at 530 °C, the photocurrent signal decays to 1 × 10^−8^ A. Photoresponse curves in Fig. 9 clearly show that the electron transport is favored by an increase of the crystallite size. According to Table 2 and the photoresponse curves from Fig. 9, a crystallite size of around 12.4 nm is necessary for building enough electron levels in both the conduction band as well as the valence band that favor the photogeneration of electron–hole pairs (*e*^−^-*h*^+^) and the transport of electrical charge.

Once the illumination has been switched off, the photocurrent signal decays in average to around 1.5 × 10^−10^ A for all photoresponse curves. From Fig. 9, it is possible to observe that TiO_2_^m^ films do not have trap states that modify the abrupt decay of photocurrent signal. That means that the electron–hole recombination is almost the same for all crystallites independently of the temperature treatment and crystal size. The photosensitivity factor *S*(= *σ*_light_ − *σ*_dark_/*σ*_dark_) of these films varies from 5.4 × 10^0^ to 7.25 × 10^2^, when the temperature increases from 350 to 400 °C and then slightly decreases to 2.75 × 10^2^ when the temperature increases to 530 °C. Those are good photosensitivity factor values considering that they are for mesoporous films. Homogeneous anatase thin films of TiO_2_ normally have a photosensitivity factor of 1 × 10^4^.

### DSSC Solar Cells

Using the TiO_2_ films prepared from pastes A127 to A210 as described in Table 2, the first step was to prepare the SnO_2_:F/TiO_2_^b^/TiO_2_^m^-type heterojunctions; then, the DSSC solar cells were assembled following the procedure described in the “Preparation of the SnO_2_:F/TiO_2_^b^/TiO_2_^m^ Heterojunction” to “The Electrolytic Solution” sections, finally resulting in a DSSC solar cell configuration of type SnO_2_:F/TiO_2_^b^/TiO_2_^m^/dye N-719/electrolyte/counter electrode/SnO_2_:F.

Once the DSSC solar cells were assembled, the experimental parameters that describe them as electrical power generating devices were determined, i.e., *V*_OC_, *J*_SC_, FF and η%.

It is convenient to remember that FF and η% are described by Eqs. 4 and 5, where *P*_MAX_ (*= V*_MAX_ ⋅ *J*_MAX_) represents the point of maximum power generated by the cell, and *V*_MAX_ and *J*_MAX_ represent the maximum voltage and current values, respectively, that maximize the power *P*_MAX_.

4 $$ \mathrm{F}\mathrm{F}=\frac{P_{\mathrm{MAX}}}{V_{\mathrm{OC}}\ {J}_{\mathrm{SC}}}=\frac{V_{\mathrm{MAX}\ }{J}_{\mathrm{MAX}}}{V_{\mathrm{OC}}\ {J}_{\mathrm{SC}}} $$

5 $$ \eta \left(\%\right)=\frac{P_{\mathrm{MAX}}}{P_{\mathrm{O}}}*100=\frac{V_{\mathrm{MAX}\ }{J}_{\mathrm{MAX}}}{P_{\mathrm{O}}}*100=\frac{V_{\mathrm{O}\mathrm{C}\ }{J}_{\mathrm{SC}}FF}{P_{\mathrm{O}}}*100, $$

where *P*_O_ (W/m^2^) represents the irradiance that reaches the solar cell. Table 3 describes the pressure values at which the TiO_2_ nanoparticles were synthesized as well as pastes used to prepare the n-type TiO_2_ mesoporous semiconductor used in the construction of the DSSC solar cells and the values for the parameters *V*_OC_, *J*_SC_, FF, and η%, which characterize the solar cells.

**Table 3:** Experimental parameters of the DSSC-type solar cells relative to the paste used to deposit the porous TiO_2_ film

Synthesis pressure (atm)	Paste	*V*_OC_ (V)	*J*_SC_ (mA cm^−2^)	FF	η%
4	A127	0.71	6.04	0.65	2.84
9.5	A148	0.73	6.75	0.68	3.38
19.5	A167	0.66	9.51	0.66	4.21
31.7	A182	0.66	9.68	0.66	4.30
54	A200	0.65	14.55	0.58	5.63
71	A210	0.67	10.50	0.61	4.36

**Fig. 10:**
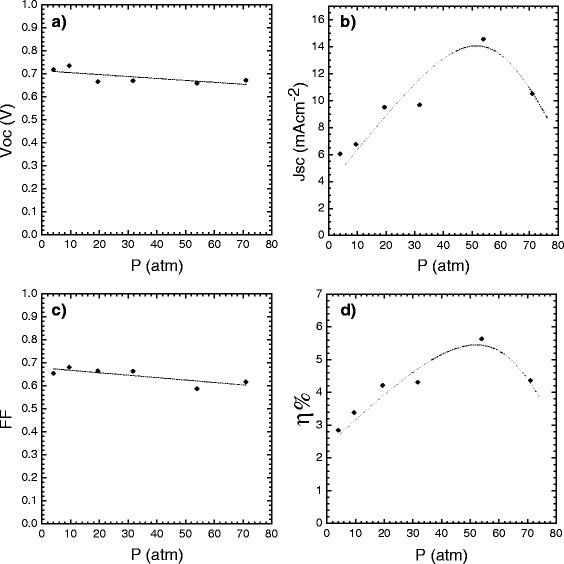
Plots of DSSC solar cell parameters: **a** *V*_OC_, **b** *J*_SC_, **c** FF, and **d** η% vs. synthesis pressure (*black diamonds*) of the TiO_2_ nanoparticles

Figure 10a–d shows the behavior of *V*_OC_, *J*_SC_, FF, and η% of the DSSC solar cells as a function of the existing pressure in the autoclave during synthesis of the nanoparticles in the hydrothermal processes. Figure 10a, it is easy to observe that the values of *V*_OC_ decrease slightly from 0.73 to 0.65 V as the pressure in the autoclave increases from 4 to 71 (atm), whereas *J*_SC_ increases from 6 (mA/cm^2^) until it reaches a maximum of 14.55 (mA/cm^2^) under the same pressure variations, as shown in Fig. 10b. Similarly, it is easy to observe from Fig. 10c that the FF values do not exhibit much variation with pressure and oscillate from 0.6 to 0.7.

Finally, the η% values (see Fig. 10d) obtained from the DSSC cells exhibited an increasing trend until reaching a maximum value of 5.63 % and then decreased when the pressure increased from 4 to 71 (atm). What stands out from Figs. 10b and 10d are the behaviors of *J*_SC_ and η%, which exhibit very similar trends and suggest that *J*_SC_ models the behavior of η% because for the same irradiance and surface area in all the DSSC cells, *V*_OC_ remains approximately constant and FF does not exhibit much variation with the different pastes. This finding suggests that reducing the impedance of the DSSC cell can increase *J*_SC_ and therefore η% of the cell.

**Fig. 11:**
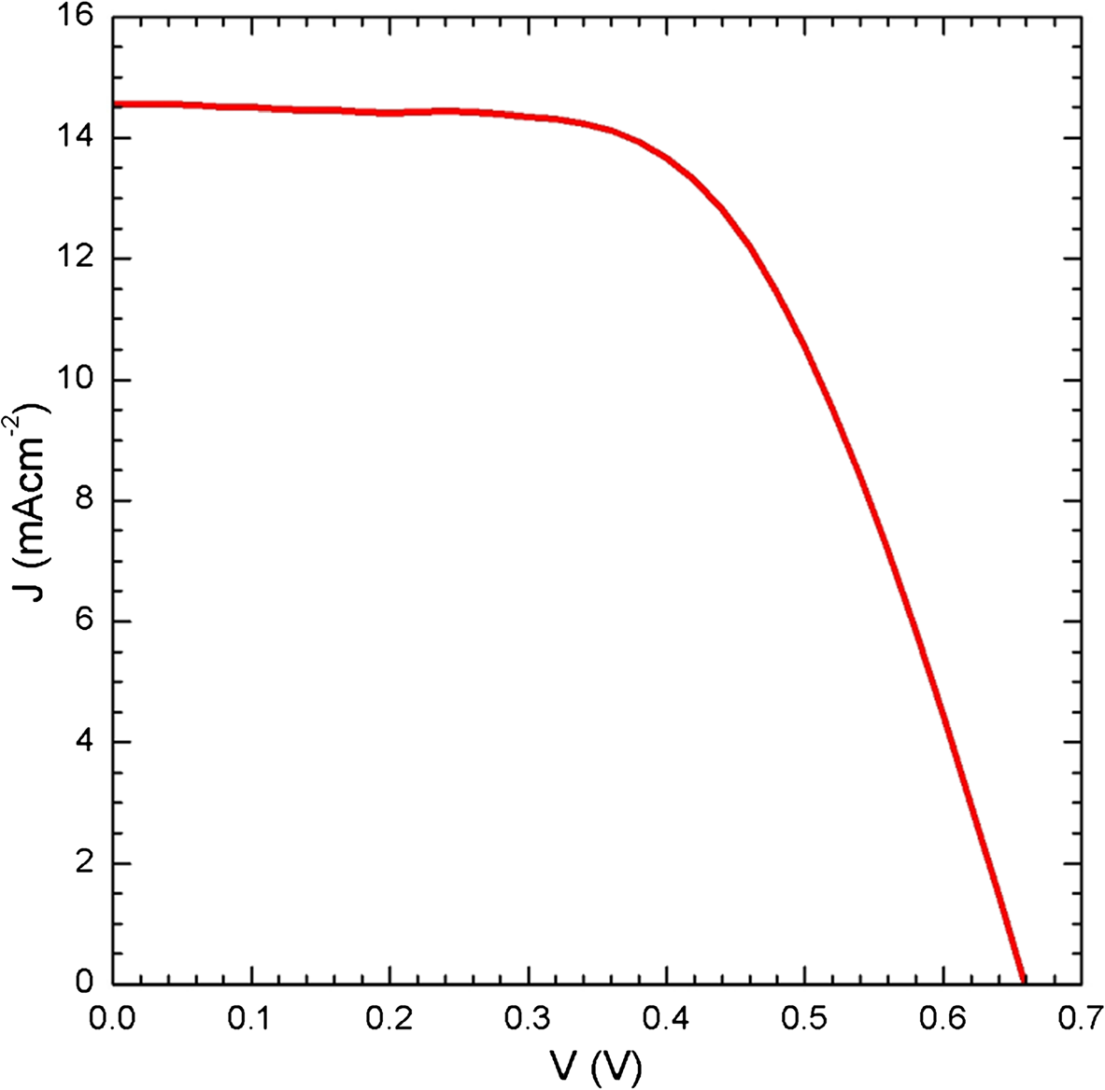
*I*–*V* curve of the DSSC-type solar cell in which paste A200 was used to prepare the TiO_2_^m^ mesoporous film, which was later sensitized using dye N-719. The DSSC cell has experimental parameters *V*_OC_ = 0.659 V, *J*_SC_ = 14.556 mA cm^−2^, FF *=* 0.587, and η% *=* 5.637 %

Figure 11 presents the *I* vs. *V* curve of the DSSC-type solar cell, which corresponds to the maximum value of the curve shown in Fig. 10d for which the paste A200 was used to prepare the mesoporous TiO_2_^m^ film as the n-type semiconductor material, which was sensitized with dye N-719. The DSSC solar cell in Fig. 11 has experimental parameter values *V*_OC_ = 0.65 V, *J*_SC_ = 14.55 mA cm^−2^, FF = 0.58, and η% = 5.63 %. Figures 10 and 11 graphically summarize the important role played by the nanostructured and mesoporous TiO_2_^m^ layer as the n-type semiconductor material in the design of the DSSC solar cells.

The optoelectronic properties of the n-type nanostructured TiO_2_^m^ prepared in our lab and the results obtained by its application in DSSC solar cells are in accordance with similar results reported by other research groups with the same semiconductor. For example, the commercial titanium dioxide Degussa P25 (80 % anatase/20 % rutile, 50 m^2^/g and 21 nm average crystal size) has been used as an n-type semiconductor layer in the development of DSSC solar cells. Kyung-Jun Hwang and coworkers achieved a 5.86 % of conversion efficiency by using Degussa P25 in the photoanode of their DSSC prototype [28]. Similarly, Chao-Chin Sua [29] and collaborators developed a 4.7 % efficiency DSSC solar cell by using the same commercial semiconductor while the research group of Yunfeng Zhao [30] reached a conversion efficiency of 7.6 % in their dye-sensitized solar cell. Additionally, the Degussa P25 (DP25) has been used as support for the growth of TiO_2_ nanorods (Tnr) with a crystalline phase of anatase in order to create a DP25/Tnr composite for the DSSC photoanode. Under this configuration, Chao-Chin Sua et al. were able to achieve a conversion efficiency of 7.56 % in their DSSC solar cell [29].

Recently, Wu-Qiang Wu and coworkers have designed new types of architectures for the n-type semiconductor. In order to achieve a bigger surface area, a better electron transport, and a higher charge collection, they prepared photoanodes with ultra-long vertically aligned multilayered anatase TiO_2_ nanowires on FTO glass substrates. This allowed them to reach conversion efficiencies in DSSC solar cells as high as 9.40 % [31]. They also developed a hierarchical assembly of macroporous material–nanowire-coated metal oxide composite electrodes with the purpose to increase the dye loading. The dye-sensitized solar cells based on a TiO_2_-macroporous-material–TiO_2_-nanowire composition electrode showed a conversion efficiency of 9.51 % [32]. On the other hand, hyperbranched array materials of well-organized nanostructures of mesoporous TiO_2_ films and well-aligned one-dimensional (1D) nanostructures, such as nanowires (NWs), nanosheets (NSs), and nanorods (NRs) grown on TCO, were also used to prepare efficient photoanodes for DSSC solar cells. The design of new hyperbranched array materials for the photoanode allowed them to reach a conversion efficiency of 9.09 % in DSSC solar cells [33]. The next step by creating well-organized nanostructures for the n-type semiconductor was the design of three-dimensional hyperbranched titania architecture as efficient multistack photoanode. This array was constructed via layer-by-layer assembly of hyperbranched hierarchical tree-like titania nanowires (underlayer), branched hierarchical rambutan-like titania hollow submicrometer-sized spheres (intermediate layer), and hyperbranched hierarchical urchin-like titania micrometer-sized spheres (top layer). Hyperbranched arrays exhibit a substantially enlarged surface area, for which a twofold increase of the dye-loading capacity is possible. The DSSC solar cell constructed with this hyperbranched architecture showed a power conversion efficiency of 11.01 % [34].

Comparing the results obtained in DSSC solar cells whose n-type semiconductor is made of TiO_2_ nanoparticles of approximately spherical shape with 1D dimensional nanostructures, it appears that a new architecture for the n-type semiconductor with a larger surface area and better electron transport provided by 1D dimensional nanostructures is the key to achieve higher conversion efficiencies in DSSC solar cells.

Throughout all this study, we observe that in a solar cell, it is very important to optimize the n-type layer of the solar cell, which allows us to assert that, likewise, it is necessary to optimize each component one by one, which would surely increase the conversion efficiency of the DSSC solar cell.

## Conclusions

Titanium oxide synthesis under a hydrothermal treatment generated samples primarily composed of the anatase phase, with crystallite sizes that increased linearly from 9 to 13.8 nm with increasing synthesis temperature. The TiO_2_ nanoparticles were used in the design of DSSC solar cells. Each layer of the DSSC solar cell plays an important role in the fundamental parameters (*V*_OC_, *J*_SC_, FF, and η%*)* that describe its performance. This article emphasizes the optimization of synthesizing the mesoporous TiO_2_ layer as the n-type semiconductor material in the solar cell. Under constant volume conditions, it was observed that the thermodynamic parameters of pressure and temperature during the hydrothermal synthesis process play important roles in the properties of the TiO_2_ nanoparticles. The crystallite size grows linearly with temperature. We highlight the fact that the crystallite size growth with pressure in the autoclave does not follow a linear relationship but instead follows the relationship *d*(nm) = 6.865[*P*(*atm*)]^0.152^, where the pressure varies from 1 to 71 atm. The pressure in the autoclave caps the crystallite size and its geometric configuration, which affects the parameters that characterize the solar cell (*V*_OC_, *J*_SC_, FF, and η%*)*.

The mesoporous n-type semiconductor material prepared based on TiO_2_ nanoparticles using the screen-printing technique exhibits a pore size between 21.40 and 26.36 nm. During the study of the direct and indirect energy band gap of the TiO_2_ nanoparticles, we detected a quantum effect in particles with crystallite sizes below 11.8 nm, whereas for crystallite sizes greater than that value, the band gap of the films exhibited a bulk behavior. Based on the study of crystal size growth as a function of pressure and temperature and on photoresponse measurements, it was found that a crystallite size of around 12.4 nm is necessary for building enough electron levels in both the conduction band as well as valence band that favor both the photogeneration of electron–hole pairs (*e*^−^-*h*^+^) as well as the transport of electrical charge.

To test the quality of the mesoporous TiO_2_^m^ n-type semiconductor material synthesized using the hydrothermal treatment, DSSC solar cells were assembled and analyzed using *I* vs. *V* curves. It was determined that the optimum thermodynamic parameters for hydrothermal synthesis of the TiO_2_ particles were *T* = 200 °C and *P* = 54 atm. The optimum nanoparticle size of the mesoporous layer of TiO_2_ was 12.4 nm, and the best η% obtained in this study was 5.63 %. The results confirm that the parameters of the TiO_2_ nanoparticle synthesis are important factors in achieving high efficiencies in DSSC solar cells.

## Abbreviations

DSSC: dye-sensitized solar cell
FF: fill factor
*J*_MAX_: maximum current density at *P*_MAX_
*J*_SC_: short-circuit current density
*P*: pressure
*P*_0_: input irradiance that reaches the DSSC
*P*_MAX_: maximum power generated by the DSSC
*T*: temperature
TCO: transparent conductor oxide
TiO_2_^b^: TiO_2_ blocking film
TiO_2_^m^: TiO_2_ mesoporous film
*V*_MAX_: maximum voltage at *P*_MAX_
*V*_OC_: open-circuit voltage
η%: conversion efficiency
